# Food neophobia and intervention of university students in China

**DOI:** 10.1002/fsn3.2575

**Published:** 2021-09-14

**Authors:** Hua Tian, Jie Chen

**Affiliations:** ^1^ College of Life Science Xinyang Normal University Xinyang China; ^2^ School of Marxism Xinyang Normal University Xinyang China

**Keywords:** China, FNS‐C, food neophobia, university students

## Abstract

**Background:**

Food neophobia was defined as the unwillingness or avoidance to eat new foods. There are many studies on food neophobia in children, but few in university students. This study was to examine the level of food neophobia of Chinese university students. The aim is to find a way to help them relieve food neophobia.

**Methods:**

A total of 2,366 university students (16–22 years old) from Xinyang normal university were recruited to conduct a questionnaire survey on food neophobia scale (FNS) of Chinese version, which contained 10 questions. Significant difference analysis and principal component analysis were conducted.

**Results:**

For Chinese university students, willingness to try new food, trust in new food, eating disorder, and food pickiness were the characteristic indexes to evaluate the food neophobia. Gender had no significant effect, but long‐term nutrition courses had a great impact on food neophobia of university students.

**Conclusions:**

The level of food neophobia of Chinese university students is relatively high. To formulate and implement a continuous diet and nutrition education plan is good and necessary to relieve the food neophobia.

**Implications for Practice:**

These data complement the limited literature on food neophobia of university students, which will help to develop intervention plans to reduce eating disorders and support the need for further research to reveal the potential mechanism.

## Graphical abstract

Investigations had revealed that the food neophobia level of Chinese university students was relatively high. The higher level of food neophobia may be related to the students from rural areas, university located in prefecture level city, low education level of their parents. Willingness to try new food, trust in new food, eating disorder, and food pickiness were the characteristic indexes to evaluate the food neophobia in university students. Gender had no significant effect, but long‐term nutrition courses had a great impact on food neophobia of Chinese university students. These data will help to develop intervention plans to reduce diet problems of university students.

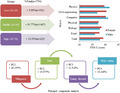

## 1 INTRODUCTION

Food neophobia defined as "unwillingness to eat and/or avoid new foods" by Pliner and Hobden (1992) is considered to be a biological mechanism, which can protect individuals from eating potentially toxic foods (Cifci et al., 2020). It is a personality trait that affects people's daily food choices (Jaeger et al., 2021). Food neophobia is related to, but theoretically distinct from picky eating (fussy eating, pickiness, finickiness) (Knaapila et al., 2015), which denotes reluctance to eat familiar foods that are not liked (Cinar et al., 2021). Food neophobia is also different from food neophobia technology. The former is afraid of unfamiliar food, while the latter is afraid of new technology(Coimbra et al., 2020).Food neophobia can refer to the actual, observable behavior, as well as the potential tendency to avoid new food. It is a suitable behavior for development in the early life span of individuals, and a persistent part of personality characteristics (Dovey et al., 2008) with considerable heredity (Faith et al., 2013) and stability (Edwards et al., 2010). Food neophobia is usually considered to be related to gender, age, personality, income, or education level. For example, male are less willing to eat new foods than female. Older people or those with lower education tend to have a higher level of food neophobia (Soucier et al., 2019). Fear related to unpleasant sensory cues of (novel) food is the key factor to refuse new food (Menghi et al., 2020). Flight et al. (2003) found that the level of food neophobia of rural adolescents was higher than that of urban adolescents, which was because urban adolescents had more opportunities to contact with different cultures and had higher socioeconomic status, thus increasing their understanding of new foods. Similarly, MacNicol et al. (2003) findings seemed to indicate that lower socioeconomic status was associated with an increased tendency to be picky about food and had a higher level of food neophobia. Olabi et al. (2009) investigated the food neophobia level of 1,122 university students in Lebanon (568) and the United States (554). The results showed that the university students in Lebanon were more averse to food than those in the United States. Cultural origin and socioeconomic status may be the source of this difference (Flight et al., 2003). Chitra et al. (2016) Studied 1,446 girls aged 15–19 in Secunderabad of southern India. The results showed that nonvegetarians had less new fear tendency than vegetarians, and they were more willing to try new/novel food and dishes. Schnettler et al. (2017) investigated 372 university students’ food neophobia and subjective well‐being in southern Chile, and found that 67.7% were classified as students with food neophobia, and family diet pattern had a significant impact on food neophobia.

As adolescents need a variety of nutritious foods for their growth (Selles et al., 2021), the World Health Organization (WHO) encourages adolescents and young people to consume five main food categories of grains, fruits, vegetables, milk and dairy products, meat, and fish. The US dietary guidelines also recommend that adolescents consume limited solid fat, cholesterol, added sugar, and refined grains. However, the actual situation is that eating habits of young people are usually described as unhealthy, with excessive intake of fat, sugar, and salt, less consumption of vegetables, fruits, poultry, and fish (Siegrist et al., 2013), and insufficient intake of protein, monounsaturated fatty acids, and dietary fiber (Meiselman et al., 1998 and Raudenbush & Capiola, 2012). Food neophobia negatively correlated with pleasantness and use frequency of fruits and vegetables and of fish and with mean pleasantness of foods (Knaapila et al., 2011). Eating disorders are common among female university students (Alshahrani et al., 2021). University Students’ compliance with the national dietary guidelines is particularly low, which indicates that the early adulthood is an important period of development that affects adults’ dietary behavior (Lowry et al., 2000). If targeted interventions are not provided in time, food neophobia can last from childhood to adulthood (Donadini et al., 2021), and then affect the diversity of food consumption of young people, as well as their willingness to change diet to support good nutrition (Raudenbush & Frank, 1999), which will bring a series of potential negative consequences (Edwards et al., 2010) affecting mental health and physical health (Schnettler et al., 2017). Therefore, this study investigated the level of food neophobia on university students in China, in order to explore the potential determinants of food neophobia, supplement the limited literature to support the need for further research, provide reference for formulating nutrition and health intervention measures in the future.

## 2 MATERIALS AND METHODS

### 2.1 Participants

All participants were native Mandarin speakers, most from Xinyang normal University, only a few of food majors from of Xinyang Agriculture and Forestry University (Table 1). Investigation was divided into two stages. Firstly, we employed 1794 university students to investigate the level of food neophobia and related influencing factors by food neophobia scale (FNS) of Chinese version (Zhao et al., 2020). Seven indicators of gender, age, nation, only child, origin, major, and parental education level were collected. Secondly, we employed 572 university students to further study the effects of gender and nutrition courses on food neophobia among university students. Students from six majors of food, biology, physical, computer, civil engineering, and physics were employed for this purpose. Data were collected through an online questionnaire created using the software Wenjuanxing (Yu et al., 2021). Participants were recruited through a snowball sampling on WeChat, a social media application widespread in China. WeChat was chosen because users are verified; thus, there is virtually zero possibilities to incur in fake profiles (Zheng et al., 2021). Researchers sent the questionnaire link to a group of WeChat teacher users and asked them to share it with their students. The questionnaire was anonymous, and participants did not receive any payment.

**TABLE 1:** Descriptive statistics of the sample

Study 1 (*N* = 1794)					
Characteristics	Frequency	Sample (%)	Characteristics	Frequency	Sample (%)
Gender			Origin		
Male	368	20.51%	Town	654	36.45%
Female	1,426	79.49%	Countryside	1,140	63.55%
Age			University Major		
16–18	609	34.11%	Liberal arts	498	27.76%
19–22	1,185	65.89%	Science	938	52.29%
Nation			Engineering course	162	9.03%
Han	1753	97.71%	Artistics	196	10.93%
Other	41	2.29%	Parental education level		
Only child			Middle school	1,286	71.68%
Yes	286	15.94%	University	495	27.59%
No	1508	84.06%	Master	6	0.33%
			Doctor	7	0.39%

Study 2 (*N* = 572)					
University major		Total	Male	Female	Nutrition courses
Food		85	51	34	√√
Biology		125	44	81	√
Physical		149	14	135	√
Computer		53	43	10	×
Civil engineering		105	40	65	×
Physics		55	19	36	×

Note

The symbol “√√” indicates that nutrition courses were offered more than one year. The symbol “√” indicates that nutrition courses were offered within one year. The symbol “×” indicates that the students had no nutrition courses.

### 2.2 Questionnaire and measures

The questionnaire we used was Chinese version of the FNS (FNS‐C). This questionnaire was translated from Food Neophobia Scale (Pliner & Hobden, 1992) by Southern Medical University, and exploration and confirmation in three samples of 1,073 healthy Chinese university students. The original questionnaire was developed by Patricia Pliner in English in 1992. The FNS‐C (Table 2) comprises 10 statements to which participants respond on a 7‐point Likert scale, ranging from 1 for “strongly disagree” to 7 for “strongly agree.” The total score is calculated as the sum of the 10 items, with five items reverse‐scored as a high score is indicative of high neophilia. Accordingly, the scale's total score varies between 10 and 70 with a single dimension. The final questions are shown in Appendix 1. Appendix 2 outlined the descriptive statistics of this sample. Excel 2013 statistical software was used for data statistics and sorting, and SPSS 20.0 data analysis software was used for significant difference analysis and principal component analysis.

**TABLE 2:** FNS‐C scores analysis (*N* = 1794)

Characteristics	FNS‐C score	FN level	Numbers	%Total(1794)	Mn ± *SD*
Total	10–70	‐	1794	100%	36.27 ± 7.61
1	10–23	Low	102	5.69%	19.72 ± 3.20
2	24–36	Middle	752	41.92%	31.34 ± 3.41
3	37–44	Middle	715	39.85%	40.10 ± 2.03
4	45–70	High	225	12.54%	48.03 ± 3.98

## 3 RESULTS

### 3.1 FNS‐C scores analysis

The FNS‐C scores of 1794 university students ranged from 10 to 70, the mean and standard deviation were 36.27 and 7.61, respectively. FNS‐C scores of the factors are shown in Appendix 3. Comparisons of FNS‐C scores and levels are shown in Table 2. Obviously, the food neophobia level of middle was up to 81.78%, accounting for the most. Among them, proportion of FNS‐C scores (24–36) was 41.92% (31.34 ± 3.41), and proportion of FNS‐C scores (37–44) was 39.86% (40.10 ± 2.03).

### 3.2 Principal component analysis

Principal component analysis (PCA) is a simplified statistical method for multivariate data, which transforms multiple indicators into a few comprehensive indicators. In order to achieve the goal of dimensionality reduction, more information should be integrated as much as possible, so the cumulative contribution rate of variance is often used to determine the number of principal components. In this study, the Kaiser–Meyer–Olkin (KMO) test showed that KMO value is 0.734, indicating that there were common factors among the items and that the scale was suitable for factor analysis. We, therefore, extracted the ten items of 1794 samples of the FNS‐C by principal component analysis to constitute a matrix of 10 × 10, then performed by SPSS20.0. After maximum variance orthogonal rotation, four factors were obtained according to the criterion of eigenvalue greater than 1. The absolute value of the factor load matrix represents the correlation between the principal component and the original variable, so the principal component can be named. In order to better explain and name variables, the load matrix of principal component is usually rotated. When the load coefficient is closer to 0 or 1, principal component can be better explained and named. They collectively explained 65.797% of the total variation (Table 3). The variance contribution rate of PC1 was 28.155%, and comprised four items (1, 4, 6, 10). The variance contribution rate of PC2 was 15.875%, and comprised two items (2, 3). The variance contribution rate of PC3 was 11.518%, and comprised one item (9). The variance contribution rate of PC4 was 10.248%, and comprised three items (5, 7, 8). All item loadings exceeded 0.50 and no double loadings were found.

**TABLE 3:** Principal component analysis (*N* = 1794)

Items	PC1 Willingness		PC2 Trust		PC3 Eating disorder		PC4 Picky eating	
	Eigenvector loading		Eigenvector loading		Eigenvector loading		Eigenvector loading	
X1	0.307	**0.751**	0.165	0.219	0.093	0.117	0.149	0.150
X2	0.026	0.082	0.506	**0.733**	−0.052	−0.066	−0.016	−0.051
X3	−0.057	−0.136	0.602	**0.836**	0.095	0.101	0.143	0.110
X4	0.316	**0.777**	−0.280	−0.103	0.056	−0.089	0.687	−0.045
X5	0.114	0.307	−0.035	0.017	−0.406	−0.470	−0.362	**−0.517**
X6	0.291	**0.714**	0.011	0.016	−0.015	−0.008	0.051	0.038
X7	0.081	0.234	0.189	0.330	−0.258	−0.301	−0.332	**−0.506**
X8	−0.113	−0.204	−0.071	−0.004	0.246	0.284	−0.642	**−0.880**
X9	0.089	0.261	0.025	0.052	0.726	**0.850**	−0.142	−0.214
X10	0.288	**0.715**	−0.116	−0.155	0.100	0.128	−0.023	0.046

Principal component		Eigenvalues		Contributions %		Cumulative contributions %		
PC1		2.816		28.155		28.155		
PC2		1.587		15.875		44.030		
PC3		1.152		11.518		55.548		
PC4		1.025		10.248		65.797		

The principal component analysis showed that four principal components of willingness to try new food, trust in new food, eating disorder, and food pickiness accounted for 65.797% of the explanatory variables, which were the characteristic indexes to evaluate the food neophobia of university students. The willingness dimension represents one's willingness to try unfamiliar food or unknown food or food from foreign countries. This factor highlights one's subjective willingness to try a new food that one has never eaten before. The trust dimension represents one's trust in food that one has never eaten. Trust is achieved through a variety of ways, such as the safety and cleanliness of food materials, the taste and nutrition of food, and other ways. This factor highlights one's fear and worry about food they have never eaten. It is similar to an emotion, such as fear or anxiety. Eating disorder is a serious mental disorder characterized by unhealthy eating habits. Female university students have the greatest risk of overeating (the early stage of eating disorder). The dimension of eating disorder represents one's incorrect eating attitude. For university students, it is mainly caused by dissatisfaction with body image of extreme body shape and weight control. The picky dimension measures whether a person is habitually picky, in order to distinguish between people who do not eat foods they have never tried before and those who do not eat specific foods even if they are familiar with them.

### 3.3 Significant difference analysis about gender

Table 4 shows the differences by gender for FNS‐C scores and distributions within tertiles. Independent sample *t*‐test showed that there was no significant difference in FNS‐C scores between male (36.14 ± 8.36) and female (36.30 ± 7.40), and among groups of low, middle, and high.

**TABLE 4:** Differences by gender for FNS‐C scores and distributions within tertiles (*N* = 1794)

	Male	Female	‐value	Total
FNS‐C score	36.14 ± 8.36	36.30 ± 7.40	*p* =.712	36.27 ± 7.61
Tertiles	*n* (%)	*n* (%)		
Low(10–23)	32 (8.70%)	70 (4.91%)	*p* =.498	5.69%
Middle (24–44)	290 (78.80%)	1,177 (82.54%)	*p* =.847	81.77%
High (45–70)	46 (12.50%)	179 (12.55%)	*p* =.219	12.54%

The conclusion is the same as Zhao et al. (2020).

As for the gender effect, some studies suggest that female tended to have fewer food neophobia than male, but the study of Southern Medical University of China also showed that there was no significant difference between male and female (Laureati et al., 2018 and Tuorila et al., 2001). Is it the same for Chinese university students? To this end, we started for the second time to further study the effects of gender and nutrition courses on Chinese university students. We employed 572 university students from six majors of food, biology, physical, computer, civil engineering, and physics from Xinyang normal university for this purpose. Why choose these majors? Because there are more male college students than female, in order to eliminate the influence of food neophobia attitude of female on male in the same class as much as possible. Significant difference analysis showed that there was no significant difference between female and male participants. The result is the same as the first time. The nutrition courses for more than one year had a great impact on food neophobia of university students (Table 5). And FNS‐C scores were lower than other students, who had nutrition courses within one year. Therefore, setting up nutrition courses is a good way to relieve or overcome food neophobia.

**TABLE 5:** Significant difference analysis of gender and majors (*N* = 572)

Item	Male (FNS‐C)	Female (FNS‐C)	t	P
Total	37.9 ± 8.72	39.22 ± 6.22	2.118	0.105
Food	36.00 ± 10.74	37.76 ± 6.57	1.815	0.073
Biology	37.86 ± 6.07	38.47 ± 6.07	1.343	0.182
Physical	37.19 ± 5.66	37.40 ± 7.54	−0.704	0.493
Computer	41.07 ± 8.84	40.19 ± 6.18	0.615	0.541
Civil engineering	36.88 ± 7.74	40.42 ± 6.16	0.759	0.450
Physics	41.63 ± 8.53	39.64 ± 7.80	0.751	0.456

Nutrition courses	University major	Biology	Physical	Computer	Civil engineering	Physics
√√	Food	0.003**	0.002**	0.083	0.177	0.189
√	Biology	‐	0.900	0.490	0.097	0.242
√	Physical	0.900	‐	0.422	0.065	0.195
×	Computer	0.490	0.422	‐	0.526	0.692
×	Civil engineering	0.097	0.065	0.526	‐	0.854
×	Physics	0.242	0.195	0.692	0.854	‐

Note

** *p* <.01. The symbol “√√” indicates that nutrition courses were offered more than one year. The symbol “√” indicates that nutrition courses were offered within one year. The symbol “×” indicates that the students had no nutrition courses.

## 4 DISCUSSION

The Food Neophobia Scale (FNS) is validated psychometric tool consisting of 10 questions. The 10‐item questionnaire used five items with positive wording and five items with negative wording. The latter were scored in reverse. These statements are measured on a 7‐point Likert scale from strong opposition to strong agreement. The higher the score, the more neophobic the individual. On the contrary, the less willing to eat new food (exotic food or novel food). The Food Neophobia Scale has been used in the United States, Canada, Australia, Finland, South Korea, and other countries, and has been translated from the original English into Swedish, Finnish, Spanish, Portuguese, German, French, and Chinese. Although there are many studies on children's food neophobia in the world, the research on university students is limited. At present, there is only one study using Chinese samples. In our study, 1794 and 572 university students from Xinyang Normal University were employed to investigate the food neophobia. All participant are about 20 years old. They have exactly left home to live independently, which is considered to be a transitional period of self‐sufficiency. They are often lack of dietary knowledge and have heavy schoolwork burden. Their daily diet is very random. They choose food according to their personal taste preference. Zhong et al. (2021) investigated the nutritional status of 263 university students in a medical university from Guangzhou in China. The detection rate of emaciation was 25.5%, the rate of overweight and obesity was 11.8%, and the detection rate of malnutrition was high. Malnutrition has been reported to affect food preferences and emotional reactions. When these lead to negative emotions, such as aversion, food‐related subjective well‐being tends to decline. As a special social group, the balanced diet, reasonable nutrition supply, and eating habits of university students are very important for their physical and mental health development. Nutrition intake in adolescence is very important for growth, long‐term health promotion, and lifelong dietary behavior development. Therefore, we investigate the food neophobia of university students, the aim is to understand their eating attitude toward new food, in order to understand their dietary status, so as to carry out nutrition intervention in time.

The average score of food neophobia in this study was a mean ±SE of 36.27 ± 7.61 (*N* = 1794), which was different with the average score of South China Medical University (33.59 ± 8.14) (Zhao et al., 2020), but consistent with that in Lebanon (36.4 ± 9.8) (Olabi et al., 2009), Southern India (37.7 ± 8.8 (vegetarian), 38.9 ± 8.3 (ovo‐vegetarian), 37.3 ± 8.6 (Nonvegetarian)) (Chitra et al., 2016), higher than that reported in developed countries, such as the United Kingdom 29.51 (26.67–30.30) (Meiselman et al., 1998), the United States (29.80 ± 11.70) (Olabi et al., 2009), Spain (31.74 ± 10.98) (Fernández‐Ruiz et al., 2013), Finnish youth (32.3 ± 10.5) (Tuorila et al., 2001), South Korea (33.50 ± 9.0) (Choe & Mi, 2011). The underlying explanations are diverse, such as coming from big cities, the number of overseas trips, the number of weekly visits to ethnic minorities, and the degree of exposure to different cultures. The less developed the country, the higher the score of food neophobia of college students. For us, the possible reason is that Xinyang Normal University is a provincial normal university located in prefecture level city. Among the 1794 research samples, the students are mainly from rural areas (63.55%), Han nationality (97.71%), non‐only‐child (84.06%), female students (79.49%) are significantly more than male students (20.51%). The major is traditional science (52.29%) and liberal arts (27.76%). The education level of their parents is low (71.68% in middle school and 27.59% in University). The future research should be aimed at the impact of sample gender ratio on FNS‐C and revealing the potential mechanism. This means that we should expand the scope of the sample and carry out the investigation of food neophobia in science and engineering universities.

## 5 CONCLUSIONS

The aim of this study is to investigate Chinese university students’ food neophobia, which is widely studied in the world, but there are only two studies in China. One is about baby, the other is about university students. The results of principal component analysis showed that four principal components of willingness to try new food, trust in new food, eating disorder, and food pickiness accounted for 65.797% of the explanatory variables, which were the characteristic indexes to evaluate the food neophobia in university students. The level of food neophobia of Chinese university students is relatively high. Gender had no significant effect on their food neophobia, but long‐term nutrition courses had a great impact on food neophobia of university students. To formulate and implement a continuous diet and nutrition education plan is good and necessary to promote their physical and mental health.

## CONFLICT OF INTEREST

The authors declare no conflict of interest.

## AUTHOR CONTRIBUTIONS

Hua Tian: Data curation‐Equal, Formal analysis‐Equal, Project administration‐Equal, Writing‐original draft‐Equal, Writing‐review & editing‐Equal. Jie Chen: Investigation‐Equal, Methodology‐Equal, Project administration‐Equal, Writing‐review & editing‐Equal.

## ETHICAL APPROVAL

This does not involve human or animal modeling.

## Data Availability

The data presented in this study are available on request from the corresponding author.

## APPENDIX 1 Food Neophobia Scale (FNS): original (English) version and Chinese version

**Table:**

items	English items	Chinese items
X1	1. I am constantly sampling new and different foods (R)	1.我会不断尝试没吃过的食物。(R)
X2	2. I don't trust new foods	2.我对没吃过的食物不太放心。
X3	3. If I don't know what a food is, I won't try it	3.我不会吃食材不明的食物。
X4	4. I like foods from different cultures (R)	4.我喜欢来自不同文化的食物。(R)
X5	5. Ethnic food looks weird to eat	5.民族特色的食物对我来说很奇怪,从而不想去吃。
X6	6. At dinner parties, I will try new foods (R)	6.在晚宴上,我会尝试没吃过的食物。(R)
X7	7. I am afraid to eat things I have never had before	7.我害怕吃以前没吃过的食物。
X8	8.I am very particular about the foods I eat	8.我对食物很挑剔/讲究。
X9	9. I will eat almost anything (R)	9.我什么食物都吃。(R)
X10	10. I like to try ethnic restaurants (R)	10.我喜欢尝试民族风味的餐厅。(R)

Note: ‘R’ stands for ‘reverse item’. This FNS‐C scale was referenced from the literature

## APPENDIX 2 Mean values and Standard Deviations of FNS‐C (N = 1794)

**Table:**

Items	Mean	*SD*	Min	Max	Cronbach's a
X1	3.47	1.425	1	7	0.618
X2	4.28	1.460	1	7	0.636
X3	5.20	1.699	1	7	0.689
X4	2.71	1.368	1	7	0.621
X5	2.65	1.432	1	7	0.626
X6	2.86	1.431	1	7	0.629
X7	3.19	1.535	1	7	0.608
X8	3.84	1.670	1	7	0.666
X9	4.83	1.764	1	7	0.671
X10	3.25	1.412	1	7	0.633

Note: *SD*, Min, Max: standard deviation, Minimum, Maximum.

## APPENDIX 3 FNS‐C scores of the factors (N = 1794)

**Table:**

Factors	FNS‐C scores (Mn ± *SD*)	Factors	FNS‐C scores (Mn ± *SD*)
Gender		Origin	
Male	36.14 ± 8.36	Town	35.92 ± 8.01
Female	36.30 ± 7.40	Countryside	36.48 ± 7.36
Age		College Major	
16–18	36.67 ± 7.71	Liberal arts	36.13 ± 8.32
19–22	36.04 ± 7.73	Science	36.69 ± 7.30
Nation		Engineering course	35.30 ± 6.94
Han	36.27 ± 7.62	Artistics	35.47 ± 7.51
Other	36.44 ± 7.15	Parental education level	
Only Child		Middle school	36.30 ± 7.51
Yes	35.75 ± 8.96	University	36.16 ± 7.71
No	36.33 ± 7.41	Master	37.00 ± 6.48
